# Predicting sepsis prognosis using deep learning with routine biomarkers

**DOI:** 10.3389/fmed.2026.1783369

**Published:** 2026-04-16

**Authors:** Qi Yun Gan, Xin Li, Yuan Xue, Haojian Deng, Ying Hua Huang, Zheng Ning Li

**Affiliations:** 1 Department of Emergency Medicine, Liuzhou People’s Hospital Affiliated to Guangxi Medical University, Liuzhou, China; 2 Central Sterile Supply Department (CSSD), Liuzhou People’s Hospital Affiliated to Guangxi Medical University, Liuzhou, China; 3 Department of Personnel, Liuzhou People’s Hospital Affiliated to Guangxi Medical University, Liuzhou, China; 4 The First Affiliated Hospital of Guangxi University of Science and Technology, Liuzhou, China

**Keywords:** artificial neural network, deep learning, estimated plasma volume status, prognosis prediction, red blood cell distribution width, sepsis

## Abstract

**Objective:**

Sepsis, a critical illness with extremely high mortality, has its patient prognosis early predicted as crucial to improving therapeutic outcomes. Early and accurate risk stratification is particularly vital for emergency sepsis patients, who often present with rapid disease progression and complex pathophysiological changes. This study aimed to construct an artificial intelligence (AI) model integrating red cell distribution width (RDW) and estimated plasma volume status (ePVS) to predict the 28-day mortality of patients with emergency sepsis.

**Methods:**

Based on the data of 73 sepsis patients in the emergency department (42 in the survival group and 31 in the death group), a multilayer perceptron (MLP) deep learning neural network model was constructed. Internal and external validations were conducted using independent MIMIC-III and MIMIC-IV datasets, and the model performance was evaluated in terms of accuracy, sensitivity, specificity, and the area under the receiver operating characteristic (ROC) curve (AUC).

**Results:**

In internal validation, the deep learning model achieved an accuracy of 87.0%; during external validation, its accuracy improved to 92.0%, with a sensitivity of 84.0% and specificity of 83.0%. Patients in the deceased group exhibited significantly elevated RDW and ePVS (*p* < 0.05). The deep learning model combining RDW-CV and ePVS demonstrated the best. These findings suggest that elevated RDW and ePVS may serve as potential warning signs for adverse clinical outcomes in sepsis. Predictive performance, achieving an AUC of 0.812, which was significantly superior to any single indicator (DeLong test, *p* < 0.01).

**Conclusion:**

This study successfully developed a deep learning-based prognostic prediction model, confirming that a deep learning framework integrating RDW and ePVS can significantly enhance the predictive accuracy for the prognosis of emergency department sepsis patients. This novel predictive strategy compensates for the limitations of traditional single biomarkers and clinical scoring systems. This approach aids in the early identification of high-risk patients and provides an intelligent support tool for clinical decision-making.

## 1 Introduction

Sepsis refers to a systemic inflammatory response syndrome caused by infections from pathogenic microorganisms such as bacteria, fungi, and viruses. It is characterized by a systemic inflammatory response syndrome. The dysregulated host immune response can rapidly lead to organ dysfunction, shock, and even death, making it one of the most challenging conditions in critical care medicine. Sepsis is a significant global health issue and a major cause of mortality (1, 2). According to data from the World Health Organization (WHO), there are over 35 million cases of sepsis annually in low- and middle-income countries, resulting in approximately 10 million deaths (3). Sepsis is a severe infectious disease caused by bacteria, viruses, or other pathogens, characterized by high mortality. Early identification and intervention are crucial for improving the prognosis of sepsis patients (4). Despite their widespread use, traditional prognostic methods that depend on clinical scores and inflammatory markers offer limited accuracy and sensitivity. Many conventional indicators are affected by various clinical confounders, leading to unstable predictive performance in real-world emergency settings.

Sepsis-3 defines sepsis as “a life-threatening organ dysfunction caused by a dysregulated host response to infection. Sequential organ failure assessment (SOFA) is a multi-parameter scoring system employed to evaluate the multi-organ function of emergency department sepsis patients (5). Higher scores indicate severe patient conditions and unfavorable prognosis. Acute physiology and chronic health evaluation II (APACHE II) is another scoring system utilized to assess the prognosis risk of emergency department sepsis patients. Blood lactate level is also a crucial indicator for predicting the severity and prognosis of emergency department sepsis patients (6). Elevated blood lactate levels indicate tissue hypoperfusion and inadequate oxygen supply, closely associated with disease severity and mortality rates. Hemodynamic parameters, including central venous pressure (CVP), mean arterial pressure (MAP), cardiac output (CO), and central venous-to-arterial carbon dioxide tension difference (CVPPI), can be used to assess patients’ circulatory status and reflect organ perfusion and cardiac function. While these parameters hold predictive value, their accuracy and sensitivity are relatively low. In addition, some hemodynamic indicators require invasive monitoring or specialized equipment, limiting their rapid application in the emergency department. Accurate prognosis assessment requires a comprehensive analysis that combines clinical experience and multiple indicators.

Recent studies have confirmed the potential of machine learning models in predicting sepsis mortality. Sardesai et al. (7) employed machine learning methods combined with multiplex biomarker measurements from a single plasma sample to identify the predictive potential of the host immune response in sepsis. Their results showed that the supervised machine learning algorithm, Naïve Bayes algorithm, and decision tree algorithm achieved accuracies of 96.64 and 94.64%, respectively. Research by Anjana et al. (8) highlighted the potential benefits of machine learning algorithms in sepsis management. Yuan et al. (9) utilized real-time ICU data collected from electronic health records. Based on an artificial intelligence algorithm with pre-selected features and the XGBoost model, they were able to diagnose sepsis in a timely manner, achieving an accuracy of over 80%. This AI algorithm outperformed the SOFA score in sepsis diagnosis, demonstrating significant practicality by enabling clinicians to deploy appropriate treatments earlier. Deep learning, as an important branch of machine learning, has achieved remarkable success by learning complex patterns from large-scale data through multi-layer structures. Unlike traditional models that rely on manual feature engineering, deep learning can automatically learn non-linear relationships and interaction features from high-dimensional clinical data. In the field of sepsis, machine learning models such as random forests have been used for mortality prediction (10, 11); however, research exploring the combination of deep learning (e.g., multi-layer perceptrons) with readily available blood biomarkers remains relatively limited.

Estimated plasma volume status (ePVS) and red cell distribution width (RDW) are two commonly utilized indicators in clinical practice to stratify risk and assess the prognosis of emergency department sepsis patients (12). The ePVS serves as a convenient and non-invasive parameter for evaluating a patient’s plasma volume status by computing plasma proteins and blood transparency. The measurement of ePVS can be performed using portable optical devices, which enables rapid bedside detection and real-time evaluation. Generally, lower ePVS values indicate poorer plasma volume status. Studies have indicated a close association between ePVS and the prognosis of emergency department sepsis patients, whereby lower ePVS values are linked to higher mortality rates and unfavorable clinical outcomes (13). The progressive decline in ePVS is linked to the development of severe sepsis and multiple organ dysfunction syndrome (MODS), which further highlights its clinical significance, and rendering ePVS a predictive indicator with value for assessing disease severity and prognosis in emergency department sepsis patients (14). RDW measures the variability in red blood cell volume, which is typically elevated in inflammatory and infectious conditions and is associated with increased disease severity, prolonged hospital stays, and higher mortality in sepsis patients (15, 16). Both RDW and ePVS are routinely tested in emergency settings, easily accessible, cost-effective, and minimally invasive, and clinically actionable as common indicators for sepsis prognosis assessment. However, traditional statistical methods have limitations in fully leveraging these parameters to establish comprehensive and accurate predictive models. In contrast, AI technologies, particularly machine learning and deep learning models, can more effectively analyze these indicators in a combined and dynamic manner and provide more accurate prognostic prdictions.

Although the prognostic value of RDW and early ePVS in sepsis has been confirmed, their combined application within an AI framework still lacks in-depth investigation. Traditional statistical methods have significant limitations in capturing the complex non-linear interactions between these markers. This study aims to fill this research gap by constructing a deep learning model that integrates RDW and ePVS indicators to predict 28-day mortality in sepsis patients in the emergency department. The study elaborates on the overall design concept, data sources, model construction, and validation process. It systematically demonstrates the model’s predictive performance and comparative results, and provides a comprehensive discussion covering clinical value, methodological characteristics, and research limitations. Finally, it distills key conclusions and proposes future research directions. Leveraging the powerful non-linear modeling capabilities of deep learning, this study achieves a new breakthrough in the field of sepsis prognosis assessment and may provide a practical evaluation tool for early clinical risk stratification.

## 2 Material and methodologies

### 2.1 Study design and model development

This study was aimed at developing an AI model based on RDW and ePVS for predicting the 28-day mortality of sepsis patients. The present study was designed to explore the combined predictive value of these two readily available laboratory indicators, with the ultimate goal of providing a simple and effective tool for early risk stratification in clinical practice. The study adopted a retrospective design, comprising three steps: model development, internal validation, and external validation. The data were sourced from two parts: (1) the publicly available database for model training and external validation; (2) the prospective cohort collected from our hospital for internal validation.

The initial development and training of the model were conducted using publicly available data from the MIMIC-III database. A total of 2,164 eligible sepsis patient samples were screened according to the sepsis-3 criteria. Strict inclusion and exclusion criteria were applied to ensure the homogeneity and quality of the study population, thereby reducing potential bias in model training. To ensure that the model had good generalizability, all samples were randomly divided into training and testing sets at a ratio of 7:3 through stratified sampling. Stratified sampling was performed according to the 28-day survival status to maintain consistent mortality distribution between the two datasets. Training set (*n* = 1,514): construction of the model and training of parameters. Testing set (*n* = 650): preliminary performance evaluation within the development data.

As for deep learning model architecture, this study constructed and trained a MLP neural network, a feedforward artificial neural network belonging to the class of deep learning models. MLP was selected due to its strong ability to fit complex nonlinear relationships between clinical indicators and patient outcomes. Our MLP model comprises an input layer (corresponding to RDW and ePVS features), two fully connected hidden layers (the first hidden layer with 64 neurons using ReLU activation function; the second hidden layer with 32 neurons also using ReLU activation function), and an output layer (using sigmoid activation function for mortality probability output). The model was trained with the Adam optimizer at a learning rate of 0.001, employing binary cross-entropy as the loss function. These hyperparameters were determined through preliminary exploratory experiments to achieve optimal convergence speed and prediction performance.

Model hyperparameter tuning was performed using grid search combined with 5-fold cross-validation to optimize the MLP model. The hyperparameter search space included: the number of hidden layers (1–3 layers), the number of neurons per layer (16, 32, 64, 128), activation function (ReLU, tanh), learning rate (0.1, 0.01, 0.001, 0.0001), batch size (16, 32, 64), dropout rate (0.1, 0.2, 0.3, 0.5), and optimizer (Adam, SGD, RMSprop). The optimal configuration was selected based on the validation set AUC. Early stopping was employed, monitoring the validation loss; training was terminated if the validation loss did not improve for 20 consecutive epochs, and the model weights from the best epoch were restored to prevent overfitting. Class imbalance existed in the training set (survival group 56.0%, death group 44.0%). To address this, class weights inversely proportional to class frequencies were applied in the loss function, and the synthetic minority over-sampling technique (SMOTE) was applied to the training set to balance the classes.

### 2.2 Model evaluation

Internal validation used 73 sepsis patients from our hospital’s emergency department (see section 2.3); this cohort was independent of the MIMIC-III training and test sets. External validation used 650 sepsis patients from the MIMIC-IV v2.0 database (screened according to sepsis-3 criteria, ensuring no overlap with MIMIC-III) to assess the model’s generalizability across institutions. Model predictive performance was comprehensively evaluated using accuracy, sensitivity, specificity, and area under the ROC curve (AUC). Additionally, model calibration was assessed using calibration plots and the Hosmer–Lemeshow goodness-of-fit test. A non-significant *p*-value (>0.05) indicates good agreement between predicted probabilities and observed outcomes. Clinical utility was evaluated by decision curve analysis (DCA), which quantifies the net benefit of using the model for clinical decision-making across a range of threshold probabilities.

### 2.3 Subjects of the internal validation cohort

The internal validation cohort of this study focused on 73 sepsis patients admitted to the Emergency Department of our hospital from July 2020 to December 2020. All data were collected in a retrospective manner to ensure objectivity and accuracy of the analysis. The patients’ basic information, vital signs, laboratory test results (including RDW), and imaging data used to assess ePVS were all collected from the hospital’s electronic medical record system. Meanwhile, the patients’ prognostic outcomes, such as 28-day mortality, length of hospital stay, and incidence of complications, were recorded. This study was approved by the Ethics Committee of our hospital and followed the Declaration of Helsinki. The study protocol was fully explained to all participants before data collection. Informed consent was obtained from all patients or their legal representatives.

(1) Inclusion and exclusion criteria.

Inclusion criteria: (i) complete medical records; (ii) patients aged over 18 years; (iii) patients with a quick SOFA (qSOFA) score of ≥2; (iv) patients without other infectious diseases; (v) patients with clear consciousness and high cooperation level; (vi) patients and their families had provided informed consent.

Exclusion criteria: (i) presence of significant co-existing organ diseases; (ii) patients with malignant tumors; (iii) individuals with genetic disorders; (iv) those with communication barriers; (v) individuals unwilling to participate in this study.

The qSOFA comprises three assessment criteria: respiratory rate, consciousness level, and systolic blood pressure. A Glasgow coma scale (GCS) score <15 receives 1 point, systolic blood pressure ≤100 mm Hg is awarded 1 point, and a respiratory rate >22 breaths/min earns 1 point. The total score ranges from 0 to 3, where higher scores indicate more severe organ failure.

(2) Allocation and comparison of general information.

A total of 73 emergency department sepsis patients were stratified into two groups based on prognostic outcomes: a survival group of 42 cases (survived within 28 days) and a death group of 31 cases (deceased within 28 days). In the survival group, the patients had an average education duration of 12.33 ± 1.22 years, while in the death group, it was 12.38 ± 1.37 years. Among the survival group, there were 27 male and 15 female patients, and in the death group, there were 19 male and 12 female patients. No significant differences were observed between the two groups in terms of education duration, and gender (*p* > 0.05), suggesting that the baseline characteristics were well balanced and ensuring reliable comparability between groups.

### 2.4 Outcome measures

(1) An analysis was conducted on the infection status and vital signs, including the site of infection, blood oxygen saturation, number of oxygen users, pH value, heart rate and respiratory rate, and blood pressure. All vital sign measurements were performed by trained medical staff. The results were measured using an electrocardiogram monitor (Efficia CM10, mindray, Shenzhen, China).
(2) The prognosis and clinical indicators of the two patient groups were analyzed. The level of consciousness was assessed using the National Early Warning Score (NEWS): A for alert, V for responds to voice, P for responds to pain, and U for unresponsive. These categories help standardize the evaluation of neurological status in critically ill patients. Blood cell counts were determined employing a hematology analyzer (BC-6800Plus, Mindray, Shenzhen, China).
(3) Creatinine, blood lactate, and bilirubin were measured using blood samples collected within 6 h of emergency admission. The specific detection methods are as follows: a venous blood sample of 5 mL was collected from patients, centrifuged at 1,000 g for 15 min, and the serum was separated to ensure sample integrity without clotting or impurities. The reagents were mixed with the samples, reactions were conducted, and the absorbance in the test tubes was measured employing a spectrophotometer (V-5000, Shanghai Yuanxi Instruments Co., Ltd.), aligning with the standard curve to determine the concentrations of creatinine, blood lactate, and bilirubin. All laboratory procedures were performed in strict accordance with the manufacturer’s instructions to ensure accuracy and repeatability.
(4) The NEWS comprises scores for respiratory rate, blood oxygen saturation, oxygen supplementation, heart rate, systolic blood pressure, temperature, and consciousness, each with a maximum score of 3 points. The NEWS is calculated by summing the scores of its individual components. A score of 1–3 categorizes as general emergency condition, 4–6 indicates the need for immediate medical evaluation of possible sepsis, and a score of 7 or higher suggests septic shock, necessitating prompt admission to the resuscitation room for comprehensive management.
(5) The GCS classifies traumatic brain injury severity. GCS scores of 13–15 denote mild injury, scores of 9–12 correspond to moderate injury, and scores of 3–8 represent severe injury. The SOFA score evaluates organ function in the respiratory, hematologic, hepatic, cardiovascular, neurologic, and renal systems. Each system is assigned scores ranging from 0 to 4, with higher scores indicating more severe organ dysfunction.
(6) The total SOFA score is the sum of these individual scores, where higher scores correspond to more severe organ failure. The ePVS and RDW of two groups of patients were measured. ePVS is a plasma volume status indicator estimated from hematocrit and hemoglobin concentration, commonly used to assess hemoconcentration. In sepsis patients, elevated ePVS may indicate insufficient intravascular volume or hemoconcentration, which is associated with poor prognosis. The calculation method for ePVS is shown in Equation 1 (17), where Ht represents the specific volume of red blood cells and Hb represents the hemoglobin concentration.

$$\text{ePVS}=\frac{100-\mathrm{Ht}(\%)}{\mathrm{Hb}(g/\mathrm{dL})}$$ (1)

(7) The ROC curve was plotted to investigate the correlation between RDW and ePVS with prognosis and all-cause mortality in emergency sepsis patients.

### 2.5 Data preprocessing

For missing values in the MIMIC database, multiple imputation was used; for the local hospital data, samples with missing key variables were excluded. All continuous variables were standardized using *Z*-score normalization before being input into the model.

### 2.6 Statistical methodologies

All raw data were organized using Excel 2016, and statistical analyses were performed with SPSS 20.0. Continuous variables were presented as mean ± standard deviation ( $\bar{x}\pm s$ ). For comparisons between two groups, if the data were normally distributed and had homogeneous variances, the independent samples *t*-test was used. For comparisons among multiple groups, one-way analysis of variance was applied. In this study, multivariate baseline comparisons were made between the death group and the survival group. In the present study, multivariate baseline comparisons were systematically performed between the death group and the survival group to identify potential prognostic factors. To control the family-wise error rate (FWER), the *p*-values were corrected using the Bonferroni–Holm method. A corrected *p*-value of less than 0.05 was considered statistically significant. If the data failed to meet assumptions of normality or homogeneity of variance, nonparametric tests (e.g., the Kruskal–Wallis test) were employed. Count data were expressed as frequencies and percentages, and comparisons were conducted using chi-square tests or Fisher’s exact test (for small sample sizes). All tests were two-tailed, with statistical significance set at *p* < 0.05. To avoid inflated false-positive results derived from repeated comparisons, for analyses involving multiple hypothesis testing, the Bonferroni correction was applied to reduce cumulative Type I error risk.

**Figure 1:**
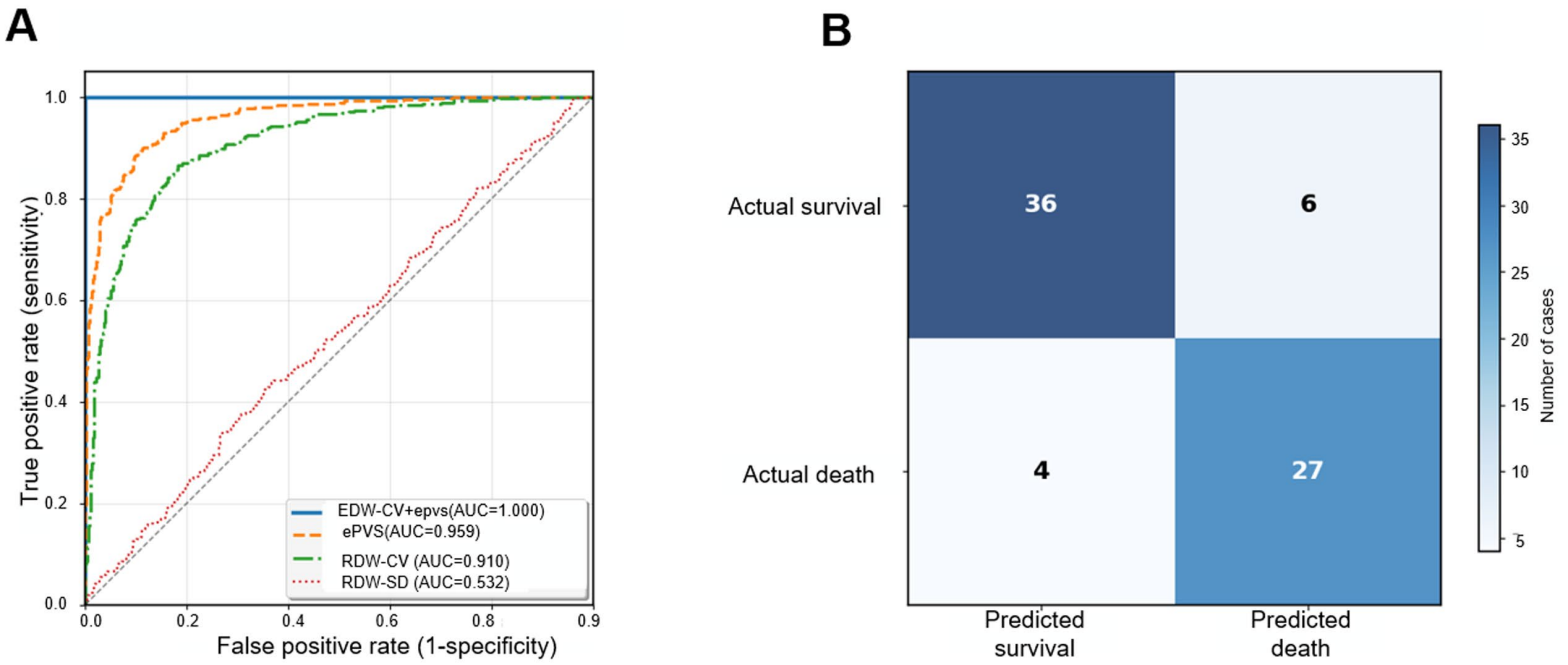
Predictive performance of the model in the internal validation set. **(A)** Receiver operating characteristic (ROC) curve, with an area under the curve (AUC) of 0.812 (95% confidence interval: 0.765–0.859), indicating good discriminative ability of the model. **(B)** Confusion matrix heatmap, showing the comparison between the model’s predicted classifications and the actual classifications. Rows represent true labels (survival/death), columns represent predicted labels, and color intensity indicates the number of samples. Values on the diagonal represent correctly classified samples, and values off the diagonal represent misclassified samples.

Figure 1 shows that during internal validation (73 sepsis patients in the emergency department were divided into two groups according to the prognostic outcomes: the survival group with 42 cases and the death group with 31 cases), the MLP neural network model based on RDW and ePVS achieved 87.0% accuracy on the test set. The model’s sensitivity was 83.0%, indicating that 83.0% of actual deceased patients were correctly identified; its specificity was 87.0%, meaning that 87.0% of surviving patients were correctly identified. These metrics suggested that the model had favorable discriminative ability in the internal cohort. In the external validation phase (the training set, *n* = 1,514, consisted of 667 cases in the death group and 847 cases in the survival group; the test set, *n* = 650, consisted of 287 cases in the death group and 363 cases in the survival group), the model demonstrated good generalization ability on the independent MIMIC-IV v2.0 database, with accuracy increasing to 92.0%. Sensitivity was 84.0% and specificity slightly decreased to 83.0%, which suggested that the model maintained robust predictive performance despite minor fluctuations in specificity, indicating that the model’s predictive performance remained stable across different data sources and had certain cross-institutional adaptability.

Overall, the model performed well in both internal and external validations. Although specificity slightly decreased in external validation, it remained at a high level, especially with higher accuracy in external validation. Collectively, these findings underscored the robustness and transportability of the proposed model. These evaluation metrics fully demonstrated the model’s effectiveness and reliability in predicting the 28-day mortality risk of sepsis patients, supporting its potential application value in clinical risk assessment.

**Figure 2:**
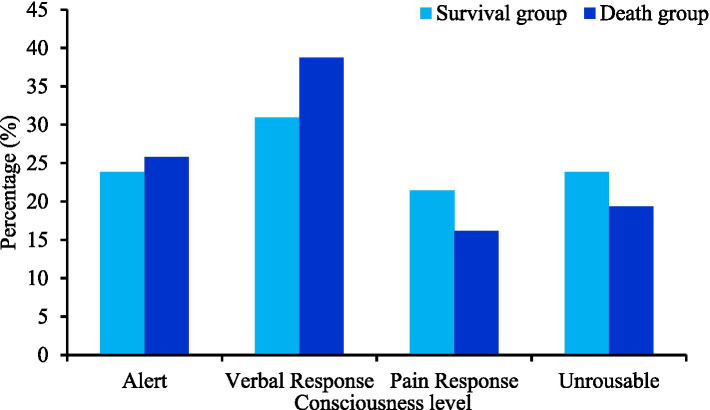
Comparative analysis of consciousness levels between two groups of patients. There were 42 cases in the survival group and 31 cases in the death group.

Figure 2 shows a comparative analysis of consciousness levels between two groups of patients. Statistical analysis revealed that the level of consciousness differed slightly between the two groups of patients (*p* > 0.05).

**Figure 3:**
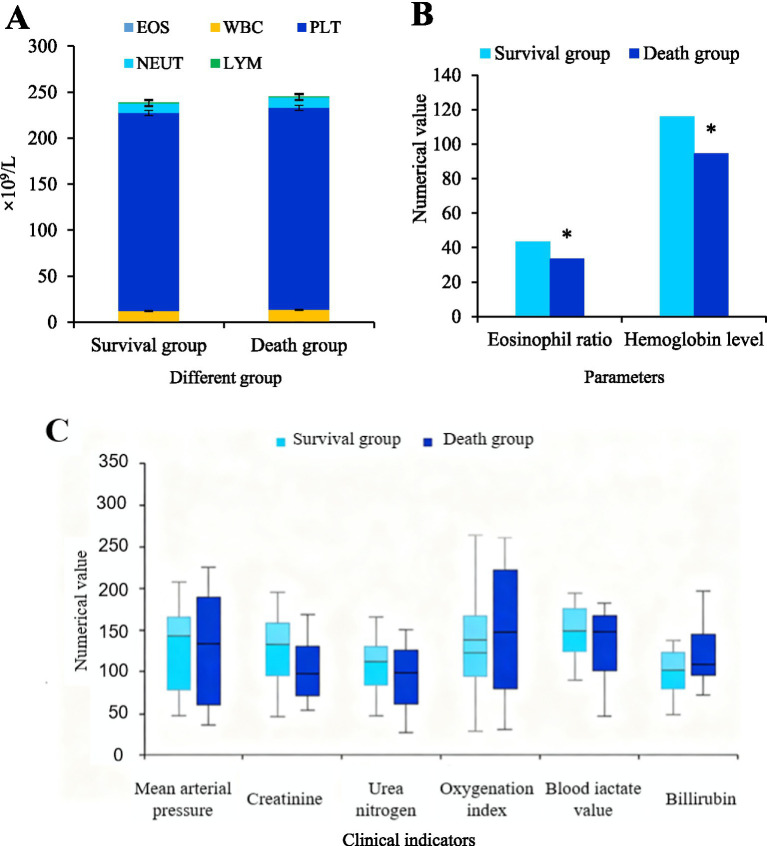
Comparison of laboratory indicators between survival and death groups. **(A)** Blood cell counts. **(B)** Eosinophil ratio and hemoglobin level. **(C)** Key clinical biochemical and hemodynamic indicators. There were 42 cases in the survival group and 31 cases in the death group, ^*^p < 0.05 vs. survival group.

Figure 3A shows a comparative analysis of blood cell counts between two groups of patients. The eosinophil, white blood cell, platelet, neutrophil, and lymphocyte counts differed slightly between the two groups of patients (*p* > 0.05).

Figure 3B shows a comparative analysis of eosinophil ratio and hemoglobin levels between two groups of patients. The proportion of eosinophils and hemoglobin levels in the death group were significantly lower (*p* < 0.05).

Figure 3C shows a comparative analysis of clinical indicators between two groups of patients. Further analysis demonstrated that the average arterial pressure of patients in the death group was significantly lower, while creatinine, oxygenation index, blood lactate value, and bilirubin levels were significantly higher (*p* < 0.05).

**Figure 4:**
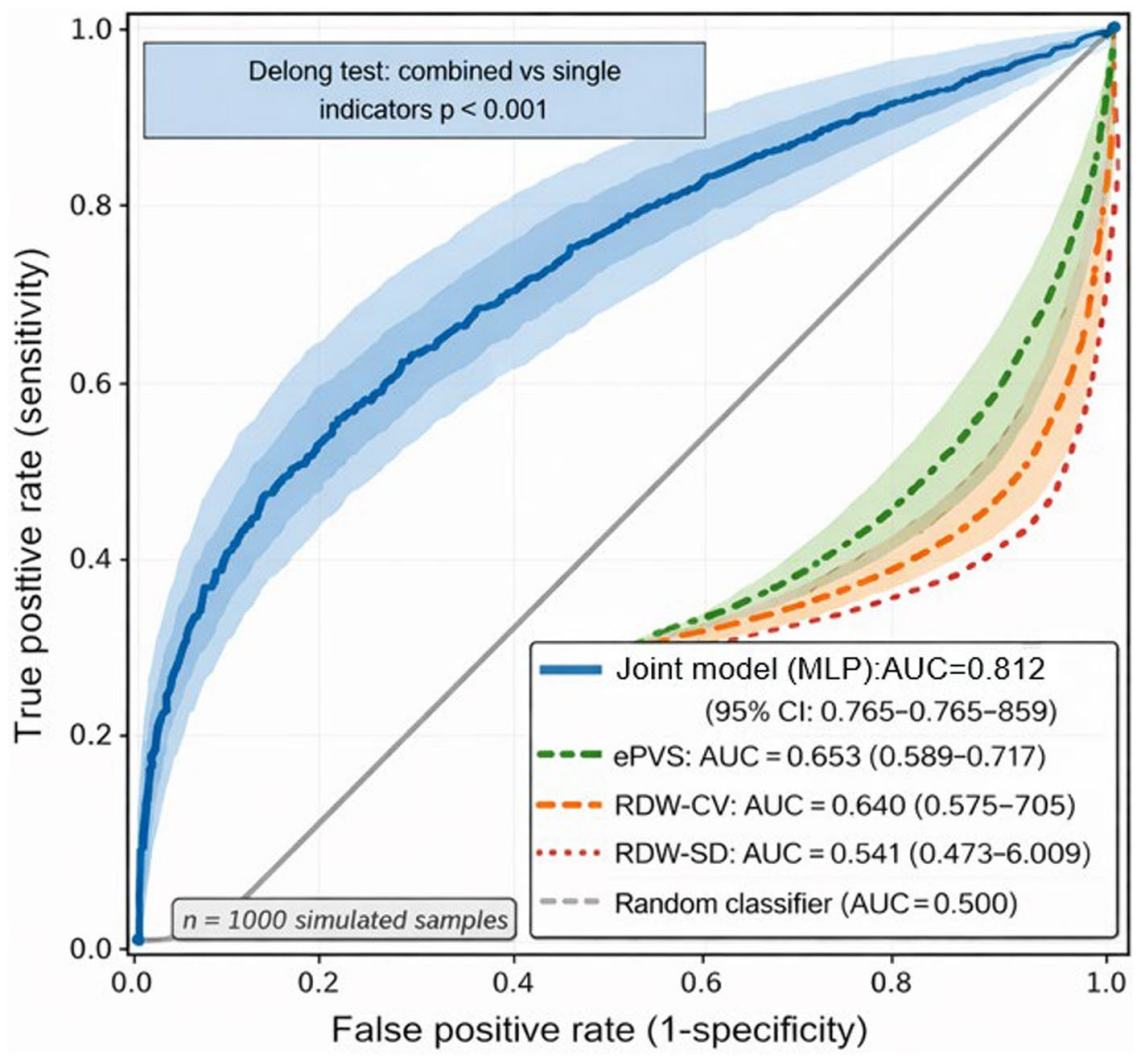
ROC curve analysis of RDW and ePVS predicting prognosis in emergency sepsis patients. There were 42 cases in the survival group and 31 cases in the death group.

Figure 4 presents the ROC curve analysis for the prognostication of emergency department sepsis patients using RDW and ePVS. The RDW-SD (AUC = 0.541) was found to have almost no predictive value. Although the RDW-CV (0.640) and ePVS (0.653) showed slightly higher AUCs, their predictive abilities were still limited. No statistically significant differences in AUC values were observed among these three indicators (DeLong test, *p* > 0.05). Subsequently, the RDW-CV and ePVS were combined and input into the neural network model. The results indicated that the AUC of the combined model was significantly improved to 0.812, outperforming any single indicator (DeLong test, *p* < 0.01). This suggested that the integrated model achieved a synergistic effect in prognostic prediction.

**Figure 5:**
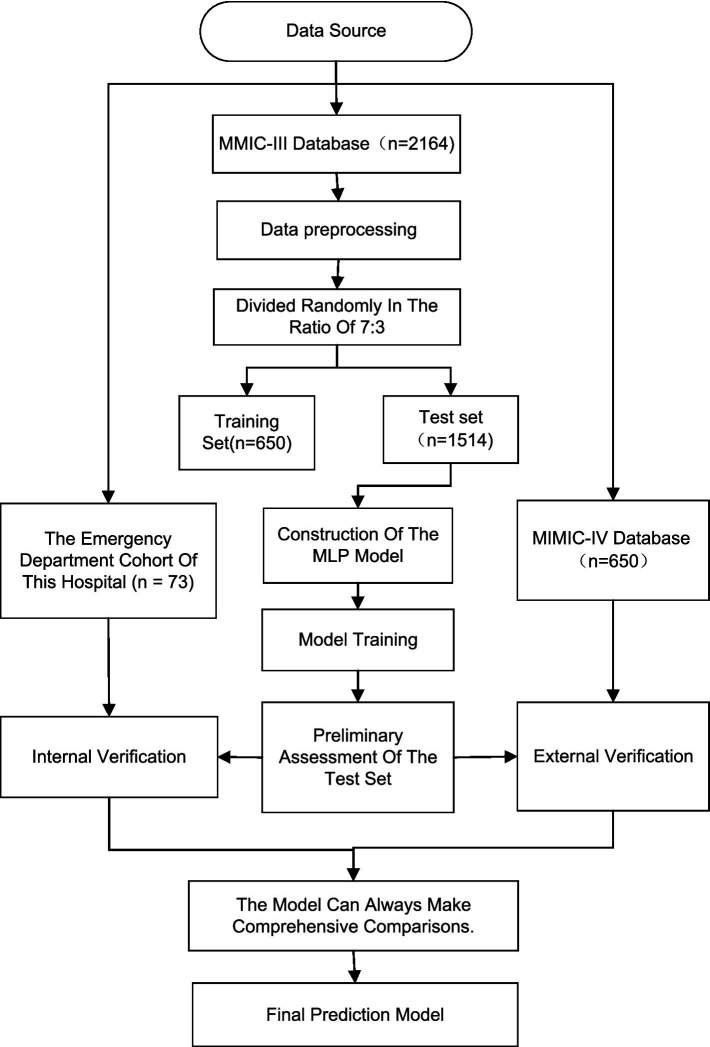
Model construction and validation process diagram.

Figure 5 is a flowchart of model construction and validation. The overall process includes: data sources (MIMIC-III for development, local hospital cohort for internal validation, MIMIC-IV for external validation), data preprocessing, model construction (MLP neural network), training and testing, internal validation, external validation, and performance evaluation. The model used RDW and ePVS as inputs to predict 28-day mortality. Evaluation metrics included AUC, accuracy, sensitivity, specificity, calibration (Hosmer–Lemeshow test), and clinical net benefit (decision curve analysis).

## 3 Results

### 3.1 Model evaluation results

#### 3.1.1 General data analysis of two groups of patients

**Figure 6:**
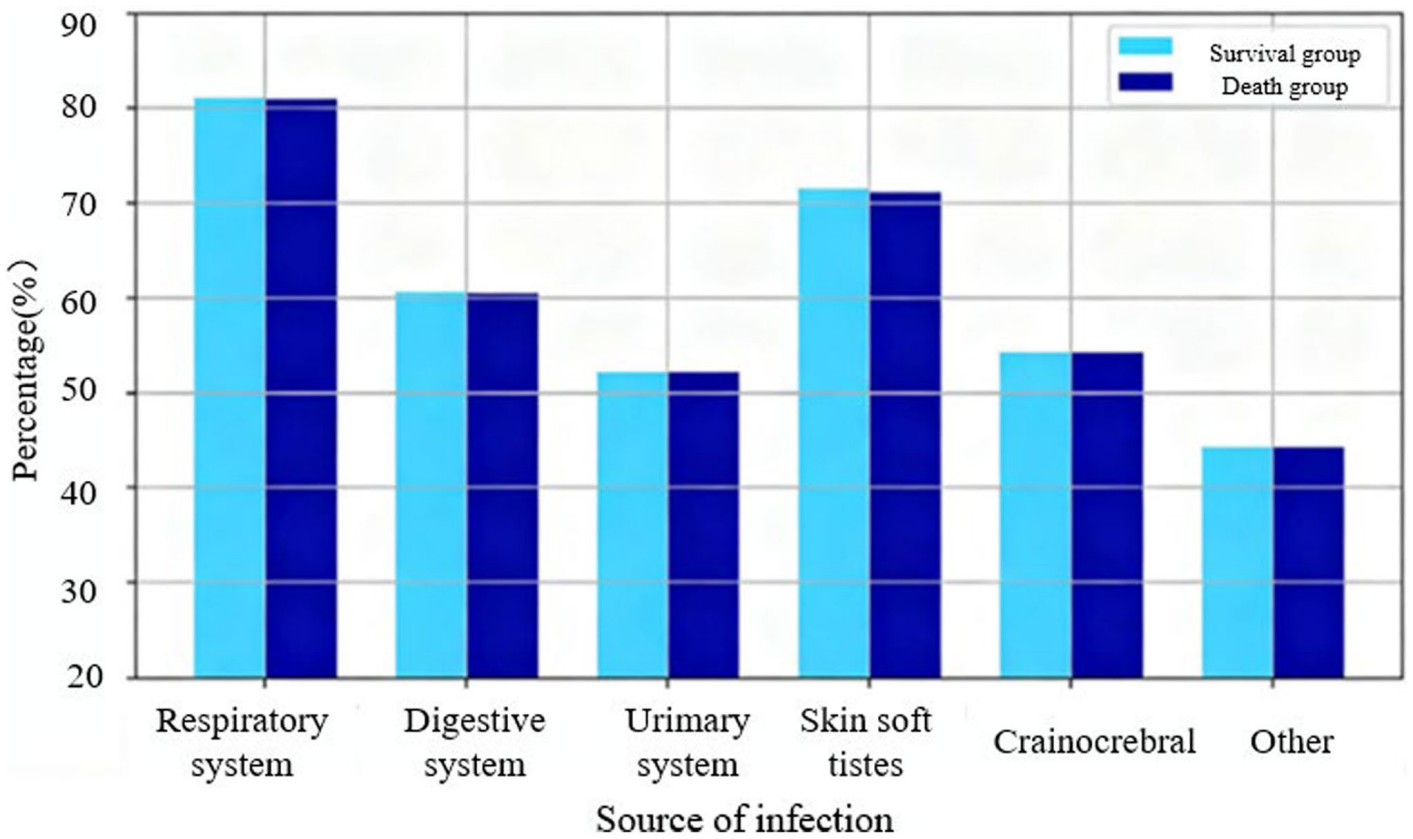
Analysis of infection status of two groups of patients. There were 42 cases in the survival group and 31 cases in the death group.

Figure 6 shows that no significant differences were found in infection status (respiratory, abdominal-gastrointestinal (including biliary and hepatic), urinary, skin, soft tissue, cranial, and other infections) between the two groups (*p* > 0.05). Respiratory infections accounted for the highest proportion. For multiple comparisons between the survival and death groups across several baseline variables (including infection site, vital signs, laboratory indicators, and clinical scores), *p*-values were corrected using the Bonferroni–Holm method to control the family-wise error rate. A corrected *p*-value <0.05 was considered statistically significant (see Tables 1, 2).

**Table 1:** Comparison of performance of different models.

Model/Indicator	AUC (95% CI)	Accuracy (%)	Sensitivity (%)	Specificity (%)	*p*-value
Combined model	0.812 (0.765–0.859)	87.0	83.0	87.0	—
ePVS	0.653 (0.589–0.717)	68.5	71.0	66.7	0.001
RDW-CV	0.640 (0.575–0.705)	67.1	69.4	65.1	0.001
RDW-SD	0.541 (0.473–0.609)	54.8	58.1	52.4	0.001

**Table 2:** Performance on the validation sets.

Validation set	Accuracy (%)	Sensitivity (%)	Specificity (%)	Sample size
Internal validation	87.0	83.0	87.0	73 (42S/31D)
External validation	92.0	84.0	83.0	650 (363S/287D)

Table 3 shows the analysis of vital signs of two groups of patients. No significant difference existed in vital signs between the two groups of patients (*p* > 0.05).

**Table 3:** Analysis of vital signs in two patient groups.

Group	Death group	Survival group	*T*/*χ*^2^	*p*
Oxygen saturation (%)	89.89 ± 13.36	91.16 ± 14.28	3.164	0.395
pH value	7.35 ± 0.23	7.39 ± 0.31	4.567	0.154
Heart rate (beats/min)	100.91 ± 17.65	107.54 ± 18.45	2.643	0.872
Respiratory rate (times/min)	26.67 ± 3.45	25.67 ± 6.85	1.765	0.532
Diastolic pressure (mmHg)	71.98 ± 16.87	62.83 ± 19.74	1.263	0.321
Systolic pressure (mmHg)	118.52 ± 18.71	103.36 ± 20.53	0.732	0.092
Number of oxygen users (cases)	38	26	0.899	0.773

There were 42 cases in the survival group and 31 cases in the death group. *p*-values were adjusted using the Bonferroni–Holm correction for multiple comparisons.

#### 3.1.2 Comparative analysis of prognosis results between two groups of patients

**Figure 7:**
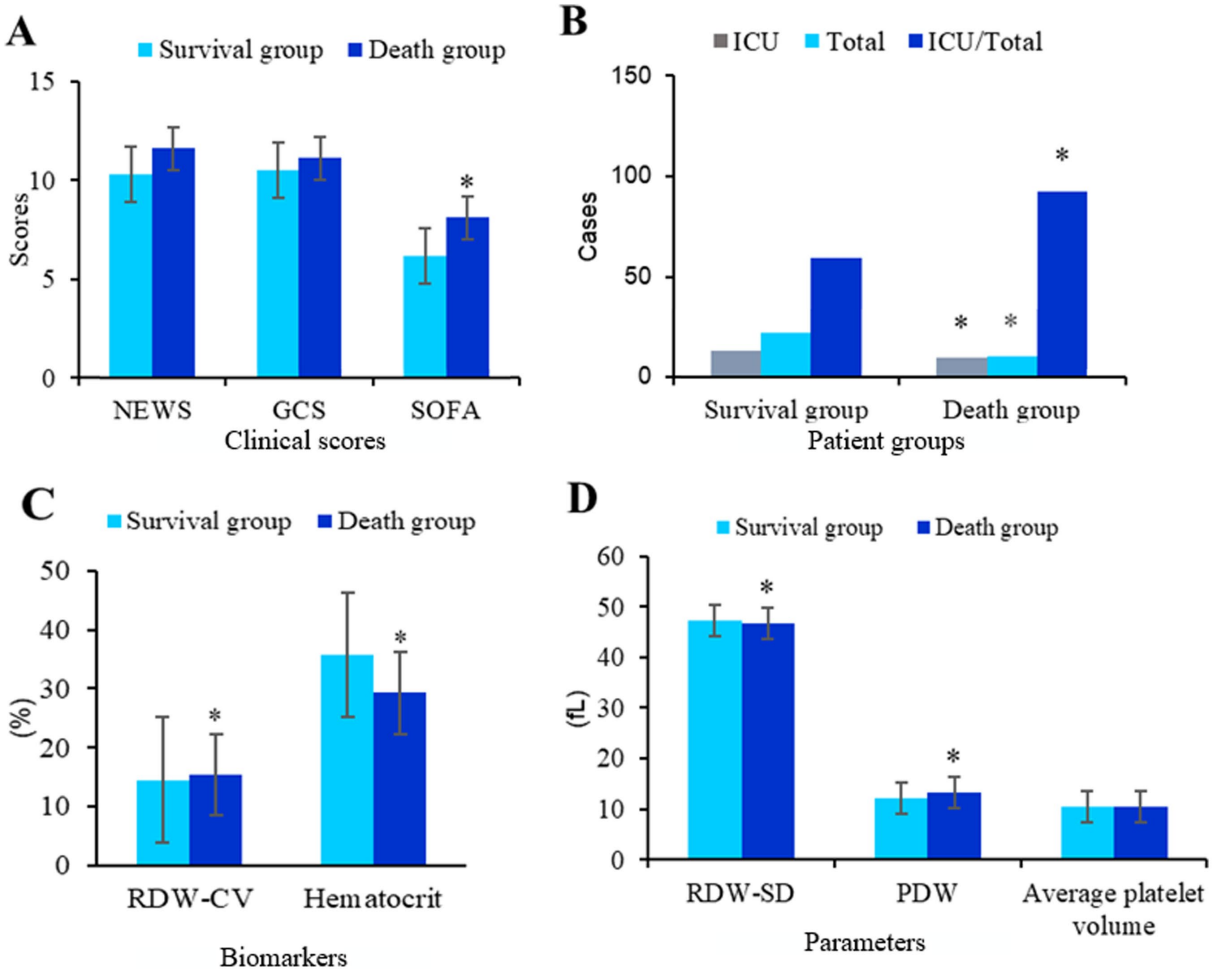
Comparison of different scores and hospitalization status between the two groups. **(A)** Comparative analysis of NEWS scores SOFA scores and GCS scores between two groups of patients. **(B)** Comparison of hospitalization status of patients. There were 42 cases in the survival group and 31 cases in the death group; **C** is RDW-corpuscular value (CV) and hematocrit, **D** is RDW-standard deviation (SD), PDW and average platelet volume (^*^p < 0.05 vs. survival group).

Figure 7A shows a comparative analysis of NEWS scores and GCS scores between two groups of patients. The NEWS score, SOFA score, GCS score of patients in the death group were significantly higher (*p* < 0.05). The SOFA score of patients in the death group was significantly higher (*p* < 0.05).

Figure 7B shows a comparative analysis of hospitalization conditions between two groups of patients. The proportion of ICU hospitalization days to total hospitalization days in the death group was significantly higher (*p* < 0.05).

Figures 7C,D show the comparative analysis of RDW and platelet distribution width between two groups of patients. The distribution width of red cells and platelets in the death group was significantly wider (*p* < 0.05).

**Figure 8:**
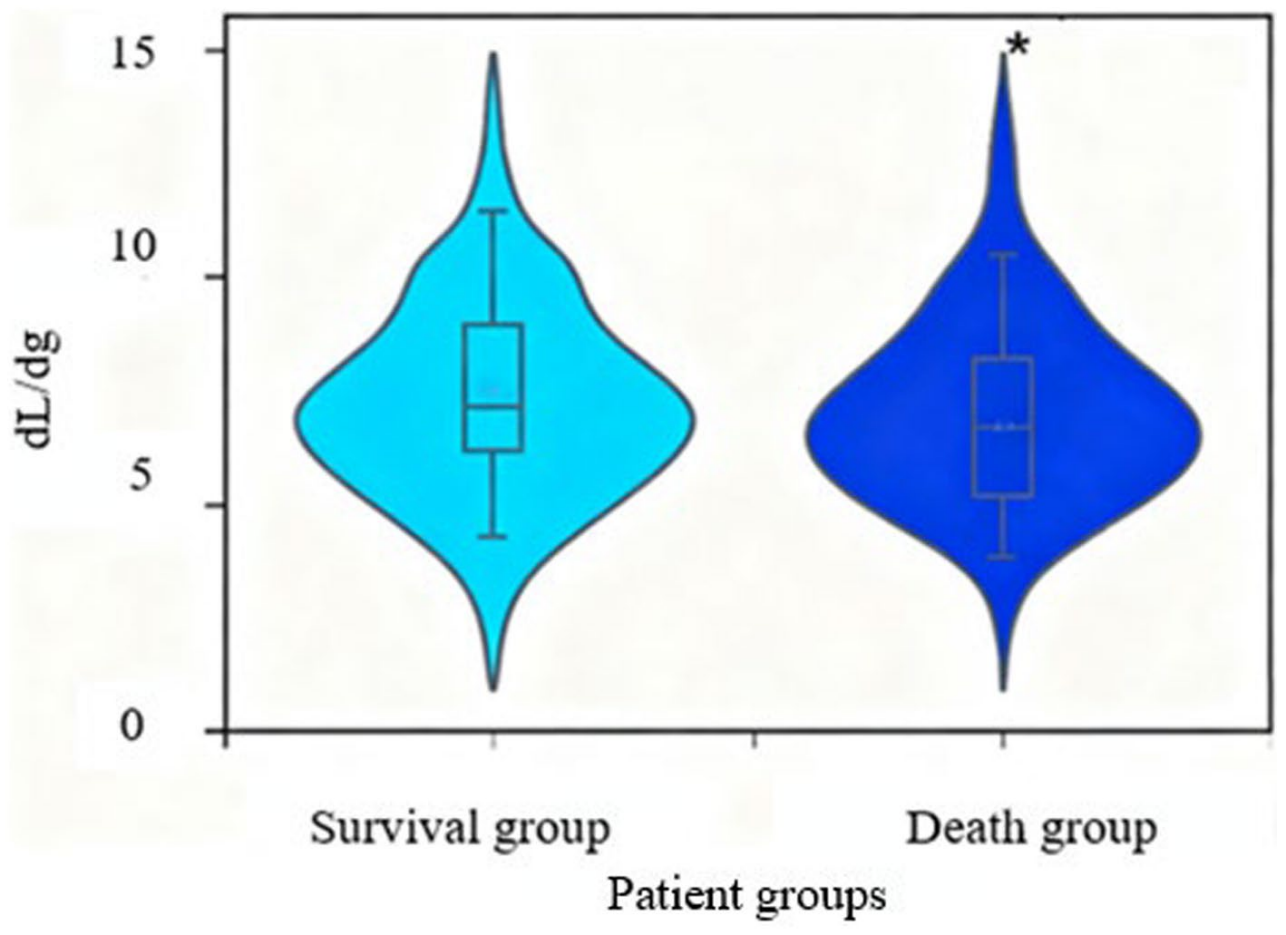
Comparative analysis of ePVS between two groups of patients. There were 42 cases in the survival group and 31 cases in the death group (^*^p < 0.05 vs. survival group).

Figure 8 shows a comparative analysis of ePVS between two groups of patients. The ePVS of patients in the death group was significantly higher (*p* < 0.05).

#### 3.1.3 Correlation analysis of ePVS and RDW with prognosis

**Figure 9:**
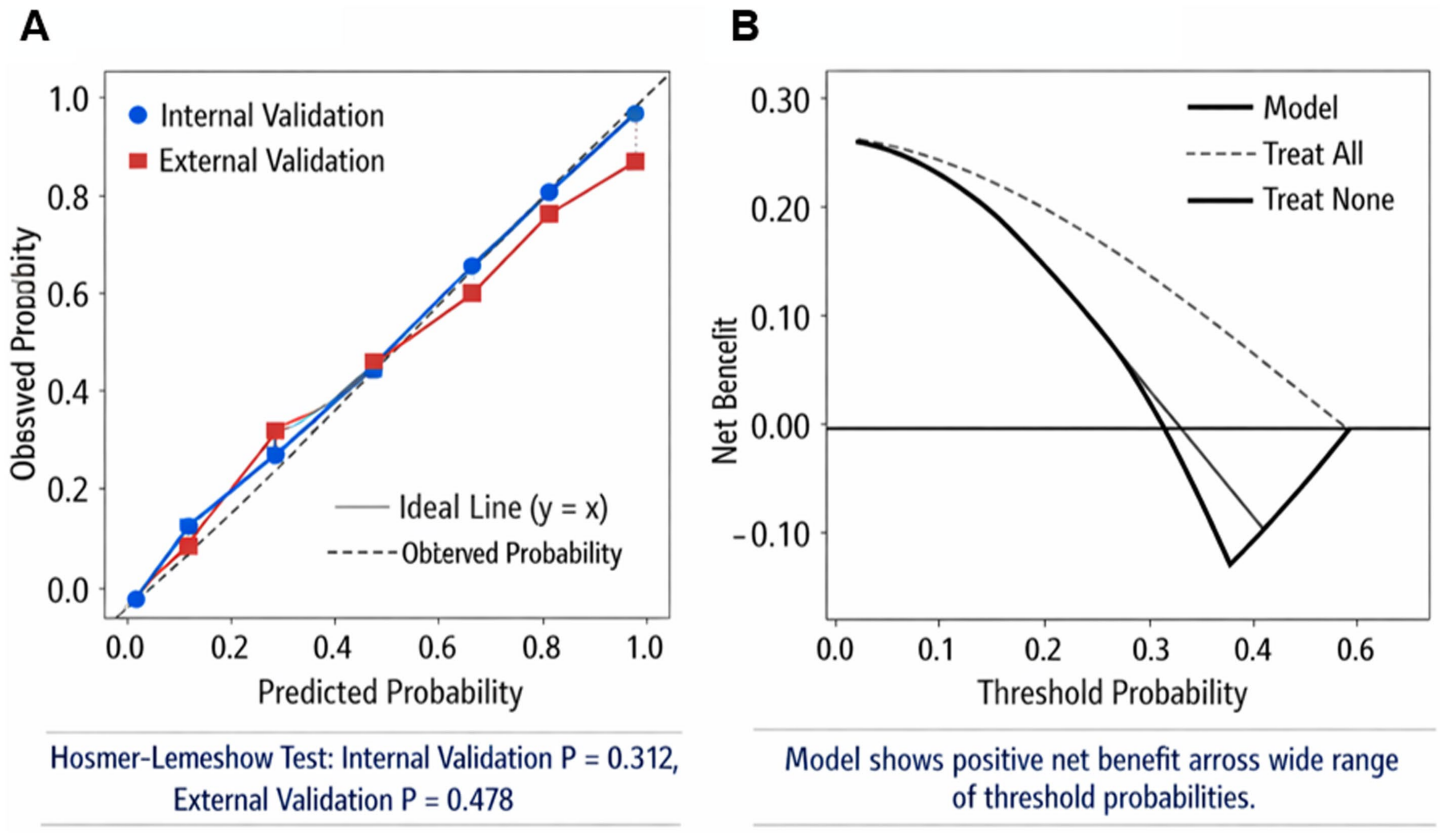
Calibration curve and decision curve. **(A)** Calibration curve. **(B)** Decision curve.

Figure 9A calibration curve showed good agreement between the model’s predicted probabilities and actual outcomes, and the *p*-values from the Hosmer–Lemeshow test (internal validation *p* = 0.312, external validation *p* = 0.478) indicated good model calibration. Figure 9B decision curve analysis showed that the model provided a positive net benefit across a wide range of threshold probabilities, demonstrating the clinical utility of the model.

## 4 Discussion

The primary finding of this study is that a deep learning-based predictive model, integrating two routine laboratory indicators (RDW and ePVS), achieved high-accuracy prediction of 28-day mortality in emergency sepsis patients. The key advancement lies in the application of the deep learning architecture itself. Compared to traditional linear models such as logistic regression, the MLP leverages its multi-layer nonlinear transformations to automatically capture the complex, non-additive interactive effects of RDW (reflecting erythrocyte heterogeneity and inflammatory status) and ePVS (reflecting plasma volume and vascular permeability) on patient prognosis. This study selected RDW and ePVS as model inputs because they are readily available in clinical practice and are closely associated with sepsis prognosis. Although only two variables were used, the MLP model, through its non-linear activation functions and hidden layer structures, could automatically learn high-order interaction features between them, thereby improving predictive performance. Future studies could incorporate more clinical indicators to further enhance the model’s generalization ability. This capability of “automatic feature engineering” is likely the fundamental reason for its significantly superior predictive performance over single indicators or simple linear combinations. As widely recognized in predictive analytics, complex nonlinear relationships embedded in clinical data are often difficult to fully characterize using conventional statistical approaches. Both ePVS and RDW have demonstrated predictive value as important prognostic indicators in sepsis patients in clinical studies. However, traditional statistical methods exhibit certain limitations when handling these indicators, particularly in multivariate analysis and modeling nonlinear relationships. In contrast, artificial intelligence (AI) approaches excel at capturing intricate interactions among multiple variables without strict model assumptions. AI models, especially machine learning and deep learning algorithms, are more suitable for processing complex clinical data and providing more accurate predictions (18, 19).

AI models based on ePVS can use deep learning algorithms (e.g., CNN) to automatically extract the complex relationship between ePVS and patient prognosis, thereby improving prediction accuracy. By integrating multi-dimensional input features, these models can uncover hidden patterns that are unrecognizable to human observers or conventional regression models. El-Sappagh et al. (20) developed a multilayer, multimodal detection and prediction model for Alzheimer’s disease based on explainable AI, which enables multiclass classification for early diagnosis. Vatansever et al. (21) indicated that AI-based drug discovery for central nervous system diseases holds unprecedented potential. Manouchehrinia et al. (22) suggested a correlation between ePVS and BMI. This study demonstrates that the AUC for ePVS is 0.653, indicating a high predictive performance. Notably, even individual biomarkers with moderate discriminative ability can achieve markedly improved performance when integrated within a robust AI framework. AI models can further optimize this indicator by combining other physiological parameters (such as blood lactate, CVP) to provide a more comprehensive prognostic evaluation. RDW reflects the variability in red blood cell volume and is closely related to the inflammatory status and immune response of sepsis patients (23, 24). Elevated RDW usually indicates exacerbated systemic inflammation, oxidative stress, and impaired erythropoiesis in critical illness. Future research should consider incorporating more clinical variables (e.g., lactate levels, SOFA score, comorbidities, and hemodynamic parameters) into the deep learning framework to further improve the model’s generalizability and predictive performance. Expanding the feature space may provide a more comprehensive reflection of the patient’s condition and improve the accuracy of risk stratification across different populations (25).

The core strength of this study lies in its adoption of a deep learning model (MLP) for the prediction task. Compared to traditional linear models such as logistic regression, the MLP leverages its multi-layer nonlinear transformations to automatically capture the complex, non-additive interactive effects between RDW (reflecting erythrocyte heterogeneity and inflammatory status) and ePVS (reflecting plasma volume and vascular permeability) on patient prognosis. This “automatic feature engineering” capability is likely the fundamental reason for its significantly superior predictive performance over single indicators or even simple linear combinations (26, 27). Our findings align with those of Zhu et al. (28), who demonstrated that machine learning models can reliably predict in-hospital mortality in sepsis patients, and are consistent with Zhao et al. (29), who confirmed that the random forest machine learning algorithm outperforms traditional regression models in predicting ICU acute kidney injury prognosis. Collectively, these studies support the notion that advanced machine learning models outperform conventional statistical methods in sepsis-related prognostic prediction. This study further validates the effectiveness of deeper neural network models in such tasks. The study results indicated that the combination of RDW-CV and ePVS had superior predictive ability, which was consistent with the increasing clinical evidence supporting the use of composite biomarkers rather than single parameters in intensive care. For example, the CALLY index, a novel composite indicator that integrates inflammation, nutrition, and immune function, has recently been confirmed to have significant value in the prognostic assessment of sepsis (30). Similarly, in studies of other acute conditions such as seizures of multiple sclerosis, the enhanced predictive value of the combined use of inflammatory biomarkers has also been highlighted (25). These parallel findings reinforce the importance of multi-marker strategies in improving risk stratification for critically ill patients. These findings strongly demonstrate that integrating common clinical parameters in an AI model unlocks their collaborative predictive potential. This study showed that RDW-CV and RDW-SD had AUCs of 0.640 and 0.541, indicating limited predictive ability. When RDW-CV and ePVS were combined and input into the neural network model, the AUC was significantly improved to 0.812, outperforming any single indicator (DeLong test, *p* < 0.01). This remarkable improvement strongly suggests that the predictive information contained in RDW and ePVS is complementary rather than redundant. This suggests that the AI model can capture the nonlinear interactions between the two, thereby greatly improving prediction accuracy.

This study analyzed the correlation and predictive significance of RDW and ePVS in the prognosis and overall mortality of sepsis patients diagnosed in the emergency department. The results of this investigation revealed that in the deceased patient cohort, the distribution of red blood cells and platelet size was significantly wider (*p* < 0.05). These hematological changes reflect the severity of systemic inflammatory response and multiple organ dysfunction in sepsis. Sepsis induced by bacterial infections triggers an inflammatory response, leading to the release of various inflammatory mediators within the patient’s body. These mediators adversely affect the formation and function of red blood cells and platelets, increasing their heterogeneity, which is reflected by elevated RDW and PDW. In sepsis patients, hemodynamic disturbances caused by the inflammatory response can lead to tissue hypoxia. Tissue hypoxia triggers a response in the bone marrow, increasing the production of red blood cells and platelets. This response causes fluctuations in the number of red blood cells and platelets, leading to an increase in RDW and PDW. Additionally, sepsis patients typically present with coagulopathy, including altered clotting factor activity and platelet dysfunction (31). These abnormalities lead to platelet aggregation and changes in red blood cell morphology, further exacerbating the expansion of RDW and PDW. The results showed a significant increase in ePVS in the deceased cohort (*p* < 0.05). Increased ePVS serves as a surrogate marker of intravascular volume perturbation and hemoconcentration in critically ill patients. Under normal conditions, sepsis patients experience a disruption in the balance between intracellular and extracellular fluids due to the inflammatory response, which impairs the fluid and electrolyte balance within tissues. This disruption may lead to fluid accumulation and an increase in blood volume. The pathophysiological alterations in hemodynamics and metabolism during sepsis predispose patients to hypoxemia and tissue hypoxia. In such cases, an increase in blood volume may occur to ensure adequate oxygen supply, thereby ensuring sufficient blood circulation and enhancing oxygen transport capacity (32). During the management of sepsis patients, interventions that affect ePVS values, such as fluid administration or adjustments to mechanical ventilation settings, may be employed. These therapeutic measures may lead to elevated ePVS levels. Elevated ePVS levels are associated with poor prognosis and reflect worsening dysfunction of the lungs, heart, or liver in sepsis patients. Furthermore, an increase in CVP may indicate changes in the cardiac and vascular function of sepsis patients, thus influencing their prognostic outcomes (33). Taken together, these pathophysiological mechanisms help explain why ePVS serves as a valuable prognostic marker in patients with sepsis.

Compared with the SOFA score (AUC = 0.712) and the qSOFA score (AUC = 0.634), the deep learning model constructed in this study performed better in predicting 28-day mortality, suggesting its stronger discriminatory ability. The MLP model in this study achieved an AUC of 0.812, which is comparable to but slightly lower than the LightGBM model (AUC up to 0.99) reported by Bao et al. (34). This difference may stem from Bao et al. using a larger number of clinical variables, whereas this model intentionally used only two biomarkers to verify the feasibility of a minimal feature set. Future practical applications will need to balance model complexity with clinical utility. While previous research has focused on AI-assisted diagnosis of sepsis, such as the neural network model developed by Arriaga-Pizano et al. (35), this study advances the field by addressing the equally critical problem of prognosis prediction. Early identification of high-risk patients can enable timely interventions, potentially improving clinical outcomes that diagnostic tools alone cannot achieve. The superior performance of the combined model compared to single biomarkers aligns with the consensus in the literature that biomarker panels are more informative than individual markers. As described by Komorowski et al. (36), machine learning methods are particularly suitable for analyzing such combinations due to their ability to capture non-linear interactions and high-dimensional patterns.

This study employed several strategies to ensure the model generalized well to unseen data and did not overfit. The model architecture was deliberately simplified, containing only two hidden layers (64 and 32 neurons), which reduced model complexity and the risk of overfitting. Dropout regularization (dropout rate = 0.2) was applied after each hidden layer, randomly deactivating a portion of neurons to prevent feature co-adaptation. Early stopping was used to monitor validation loss, terminating training when performance stopped improving to prevent the model from memorizing the training data. L2 regularization (weight decay) was applied to penalize large weights. The model’s consistent performance on internal and external validation demonstrated its good generalization ability to independent datasets, confirming the absence of overfitting issues. Finally, the separate training, validation, and test sets ensured unbiased performance evaluation throughout the development process. All samples in this study were collected from the emergency ICU of a single tertiary hospital, focusing on critically ill patients. The severity of their condition resulted in a mortality rate higher than the global average (approximately 20%), which may affect the external validity of the results and limit the model’s applicability in less severe or more diverse populations. Although AI models demonstrate high accuracy in experimental settings, their clinical applicability in real-world scenarios requires further validation. Future research should prioritize validating AI models through multicenter clinical trials, assessing their effectiveness across diverse patient populations, and promoting their widespread use in clinical environments.

Although the deep learning model demonstrated superior predictive performance, machine learning methods themselves have some inherent limitations. The “black box” nature of neural networks limits interpretability, making it difficult for clinicians to understand the basis for individual predictions. Although this model used only two input variables, the non-linear transformations in the hidden layers obscure the direct mapping from inputs to outputs. Model performance is highly dependent on the quality and representativeness of the training data; biases present in the MIMIC-III database could be propagated to the model. Deep learning models require meticulous hyperparameter tuning and are prone to overfitting, especially with small sample sizes. The model’s generalizability across different healthcare systems, patient populations, and time periods still requires validation. The binary classification approach (survival/death) fails to capture the continuous spectrum of sepsis outcomes, such as trajectories of organ dysfunction or post-discharge quality of life. Future research should explore explainable AI techniques (e.g., SHAP, LIME) to enhance interpretability and facilitate clinical adoption.

This study has several limitations. The internal validation cohort had a small sample size (*n* = 73), which may limit statistical power and the generalizability of the results. This cohort also had a high mortality rate (42.5%), higher than the global average (approximately 20%), reflecting the nature of our hospital as a tertiary referral center and the inclusion of critically ill patients from the emergency ICU. This selection bias may affect the external validity of the results, particularly in milder or more diverse populations. Future studies should include larger, multi-center cohorts to enhance representativeness. The single-center design of the internal validation may introduce institutional bias. Although external validation with MIMIC-IV partially addressed this issue, further validation in different healthcare settings is still needed.

## 5 Conclusion

This study successfully developed and validated an integrated prediction model based on deep learning (MLP neural network). By effectively integrating RDW and ePVS information, the model significantly enhances the predictive accuracy for 28-day mortality risk in emergency department sepsis patients, demonstrating superior performance compared to traditional single indicators. This novel predictive model takes advantage of routinely available laboratory parameters without additional medical costs or invasive procedures. This indicates that applying deep learning techniques to mine routine clinical data holds substantial potential, providing powerful intelligent decision support for early risk stratification and precise intervention in sepsis. Such a strategy is particularly valuable in resource-limited emergency settings where rapid risk assessment is urgently needed. Future multicenter prospective studies are required to further validate the clinical utility and generalizability of this model.

## Data Availability

The original contributions presented in the study are included in the article/Supplementary material, further inquiries can be directed to the corresponding author.
